# Rate of use and effectiveness of oseltamivir in the treatment of influenza illness in high‐risk populations: A systematic review and meta‐analysis

**DOI:** 10.1002/hsr2.241

**Published:** 2021-02-10

**Authors:** So‐Jung Shim, Mei Chan, Louisa Owens, Adam Jaffe, Bernadette Prentice, Nusrat Homaira

**Affiliations:** ^1^ Discipline of Pediatrics, School of Women's and Children's Health, Faculty of Medicine University of New South Wales Sydney New South Wales Australia; ^2^ Respiratory Department Sydney Children's Hospital Randwick Sydney New South Wales Australia

**Keywords:** heart diseases, human, influenza, lung diseases, oseltamivir

## Abstract

**Background:**

Oseltamivir is recommended in the treatment of influenza illness in high‐risk populations, including those with chronic heart and lung diseases.

**Objectives:**

We conducted a systematic review and meta‐analysis to determine the rate of use and effectiveness of oseltamivir in these groups of patients.

**Methods:**

The protocol for the systematic review was registered on PROSPERO (CRD42019125998). Medline, EMBASE, Cochrane CENTRAL, and CINAHL were searched for observational studies and randomized controlled trials published up to 16 February 2020. Quality appraisal of final studies was conducted using GRADE guidelines. Data were extracted using a predeveloped template. Main outcomes measured included the rate of use of oseltamivir for influenza‐like‐illness and its effectiveness in reducing disease severity in patients with cardiopulmonary diseases. Outcomes measured for effectiveness were influenza‐related complications (respiratory infections and asthma exacerbations), hospitalization rates, and time to freedom from illness. Risk of bias was assessed using Cochrane's Risk of Bias 2.0 tool for randomized trials and Cochrane's Risk of Bias in nonrandomized Studies of Interventions tool for nonrandomized trials. Where data were available, pooled analyses were conducted. Dichotomous variables were evaluated using the Mantel‐Hansel method. A random effect model was applied. Summary measures were reported as risk ratios where relevant.

**Results:**

Our systematic review identified nine studies. Oseltamivir use ranged from 25% to 100%. When oseltamivir group was compared to placebo, rates of respiratory tract infections reduced by 28% (RR = 0.72, 95% CI = 0.59‐0.90), hospitalization reduced by 52% (RR = 0.48, 95% CI = 0.28‐0.80) and median time to illness alleviation decreased by 10.4 to 120 hours. There was no significant reduction in asthma exacerbation rates.

**Conclusions:**

Our systematic review suggests that the use of oseltamivir is beneficial in reducing disease severity, however, its use in high‐risk population remains suboptimal.

**Abbreviations**
CI: confidence interval
ICD‐9‐CM: international classification of disease, ninth revision, clinical modification
ICU: intensive care unit
ILI: influenza‐like illness
RR: risk ratio
WHO: World Health Organization

## 1 INTRODUCTION

Influenza is a significant contributor to the global burden of disease causing severe illness in an estimated 3 to 5 million people^1^ and 291 243 to 645 832 respiratory deaths globally annually due to influenza‐related complications.^2^ Certain populations are at increased risk of complications due to influenza infection, including individuals under the age of 5, over the age of 65, people with medical conditions, including cardiac and respiratory disease, and pregnant women.^3^, ^4^ Patients with chronic respiratory conditions are particularly at increased risk of influenza‐related hospitalizations, need for intensive care unit (ICU) admission and ventilation when compared to those without such conditions.^5^ Influenza infection has also been associated with exacerbation of underlying respiratory diseases such as asthma and cystic fibrosis.^6^, ^7^ Furthermore, in patients with cardiac disease, there is at least two to five times increased risk of mortality from influenza.^5^

Currently, antiviral medications are the only treatment available for influenza infection. There are three classes of antiviral drugs that target influenza: the adamantanes (matrix‐2 [M2]‐ion channel inhibitors), neuraminidase inhibitors, and most recently, the selective inhibitor of influenza‐cap dependent endonuclease which is currently only approved for use in the United States of America (USA) and Japan. The adamantanes are no longer first‐line treatment for influenza due to the increasing development of resistance to these antivirals.^8^ Neuraminidase inhibitors such as zanamivir (Relenza) and oseltamivir (Tamiflu) are more widely prescribed in the treatment of influenza.

Although in 2017, the World Health Organization (WHO) downgraded oseltamivir from a “core” drug to a “complementary” drug in its list of essential medicines^9^ based on the unclear evidence around the effectiveness of oseltamivir, it is recommended that oseltamivir or zanamivir is used empirically in high‐risk populations with influenza‐like illness (ILI), even when presenting with uncomplicated disease.^3^

Oseltamivir is the drug of choice for the treatment of influenza for people aged ≥1 year due to its easy oral administration whereas zanamivir is recommended for people aged >5 years which is administered through intravenous route or inhalation.^3^ Oseltamivir is generally well tolerated and safe for use in both adults and children, with some side effects.^10^ Oseltamivir use is recommended within 48 hours of symptom onset in the patient, however, multiple studies have reinforced that earlier administration of oseltamivir results in better outcomes.^11^, ^12^, ^13^

Despite being recommended for use in ILI, rates of use of oseltamivir often remain suboptimal. Prior to the 2009 H1N1 influenza pandemic, antiviral prescribing rates in hospitalized patients were less than 30%.^14^ However, during the 2009 H1N1 influenza outbreak, prescribing rates exceeded 80% in hospitalized patients.^14^ More recent data from the 2012 to 2013 influenza season from outpatient care settings showed that <20% of high‐risk patients for whom antiviral treatment was appropriate were actually prescribed antiviral medication, with particularly low prescription rates in children.^15^

Previous studies on the effectiveness of oseltamivir have generally focused on healthy adults and children, with very few studies in high‐risk populations, especially children. Given the increased susceptibility of patients with cardiopulmonary conditions to influenza complications, research on the effectiveness of oseltamivir in this specific population may help in guiding clinical practice regarding the use of oseltamivir in this population presenting with ILI.

We conducted a systematic review and meta‐analysis to ascertain the rate of oseltamivir use and its effectiveness in people of all ages with cardiopulmonary conditions.

## 2 METHODS

### 2.1 Protocol and registration

The protocol for the systematic review was registered on PROSPERO (CRD42019125998).

### 2.2 Eligibility criteria

Study types: Randomized controlled trials and observational studies (including case‐control studies, cross‐sectional studies, longitudinal studies, and cohort studies) published up to 16 February 2020 were included. No publication restrictions were imposed. Only papers published in English language were included in the search.

Study participants: Study participants were people of any age with a chronic lung or cardiac disease (including asthma, cystic fibrosis, bronchopulmonary dysplasia, and coronary artery disease) with influenza or ILI.

Study intervention: The intervention was the use of oseltamivir for influenza or ILI in people with chronic lung or cardiac diseases. The comparison group was people with chronic lung or cardiac diseases who did not receive oseltamivir for influenza or ILI.

### 2.3 Outcome measures

Outcomes measured were the rate of use and the effectiveness of the intervention. We determined the rate of use as the rate of prescription of oseltamivir in patients with cardiopulmonary conditions. Effectiveness of oseltamivir was determined as the effect on severity of illness (measured as area under the symptom curve), rates of hospitalization, asthma exacerbations, respiratory tract complications such as tracheitis, bronchitis, pneumonia, nasosinusitis, and pharyngitis, and time to alleviation of illness measured in hours. Time to alleviation was defined differently in different studies as median or mean time to resolution of fever (temperature <37.2°C) and symptoms (chills and myalgia).

### 2.4 Information sources

Extensive search using predefined Medical Subject Heading (MeSH) terms (Appendix) was conducted in Medline, EMBASE, CINAHL, and Cochrane Controlled Register of Trials (CENTRAL). MeSH terms used include: “chronic lung disease,” “asthma,” “cystic fibrosis,” “lung dysplasia,” “heart disease” and restricted to “oseltamivir,” “Tamiflu,” “influenza,” and “influenza‐like‐illness.”

### 2.5 Study selection

Records generated by the literature search were managed using EndNote X9.^16^ Once duplicates were removed, secondary articles such as systematic reviews, literature reviews, meta‐analyses, case reports, and conference abstracts were excluded based on screening of the titles. Abstracts of all remaining articles were then screened based on PICO criteria. The full text of the remaining articles was assessed based on predetermined eligibility criteria.

The reference lists of pertinent systematic reviews and meta‐analyses identified in the search as well as reference lists of articles were also searched for relevant studies.

One reviewer (SS) conducted the initial search and screening of articles. Any ambiguities in study selection were resolved by discussion with another reviewer (NH).

### 2.6 Data collection process

Data from the systematic review were extracted using predeveloped data extraction template (Table A2). The following information was extracted from eligible studies: study duration, study design, number of participants in the study with cardiopulmonary conditions, age range of participants, clinical setting, inclusion and exclusion criteria, rate of oseltamivir prescription, method of confirmation of influenza infection, timeframe of treatment initiation, dosage of treatment administered and outcomes measured (Tables 1 and 2).

**TABLE 1:** Characteristics of studies included to evaluate the rate of use of oseltamivir

Author (year)	Country, years	Study type, design	Study size	Age of participants	Clinical setting	Inclusion criteria	Exclusion criteria	Oseltamivir (n = sample size)	No‐oseltamivir (n = sample size)	Rate of use (%)	Confirmation of influenza infection	Time to treatment initiation from symptom onset
Al Subiae (2012)	Saudi Arabia, July to December 2009	Prospective cohort study	14^a^	0 to 12 years	University hospital in Saudi Arabia	Children (aged 0‐12 years) who required hospitalization due to confirmed influenza A (H1N1)^a^		14^a^	0	100	Reverse transcriptase polymerase chain reaction (RT‐PCR)	Not stated
Bueno (2013)	Madrid, September 2010 to June 2012	Retrospective study	83a^a^ ^,^ ^b^	0 to 13 years	10 public hospitals in Madrid	Children (0‐13 years) hospitalized with influenza‐confirmed infection^c^	Risk factors for serious disease (not including asthma), nosocomial influenza infections, ICU admissions direct from emergency department, treatment after 48 hours of admission	34	49	41^d^	Rapid diagnostic test or polymerase chain reaction in nasal wash	Within 48 hours of admission
Coffin (2011)	USA, October to April 2001 to 2007	Retrospective cohort study	228^e^	0 to 21 years	Intensive care unit (ICU) of 41 pediatric hospitals in USA	Children admitted to an ICU for treatment of influenza	Antiviral medication other than oseltamivir, hospitalisation in previous 14 days	56^e^	172	25	ICD‐9‐CM diagnosis code of influenza (487.0, 487.1 or 487.8)	Within 24 hours of admission
Piedra (2009)	MarketScan databases (Thomson Reuters, Cambridge, MA) from six influenza seasons (October 1‐March 31) 2000 to 2006	Retrospective study	4550	0 to 17 years	Outpatient prescription data	Children and adolescents at high risk of influenza complications	Pneumonia diagnosis, hospitalised or residing in long‐term care facility	1401^e^	3149	31	ICD‐9‐CM diagnosis code of influenza (487.xx) applied to database	Within 1 day after influenza diagnosis

^a^ No separate data for children with CLDs, however, there was sub‐group data for children with asthma which has been used here.

^b^ Total number of children involved in the study was 287, however, children with a history of asthma was 83.

^c^ The inclusion criteria was children without any underlying conditions but asthma was not considered an exclusion criteria, so sub‐group data on children with a history of asthma were available.

^d^ Criteria for treatment varied between hospitals (some hospitals had ALL hospitalised children treated, others only with risk factors).

^e^ This data represents the sub‐group data: cardiac conditions and respiratory conditions.

**TABLE 2:** Characteristics of studies included to evaluate the effectiveness of oseltamivir

Author (year)	Country, years	Study type, design	Study size	Age of participants	Clinical setting	Inclusion criteria	Exclusion criteria	Intervention	Control	Outcomes	Confirmation of influenza infection	Treatment initiation	Treatment
Johnston (2005)	Multinational study over two influenza seasons (1998‐1999)	Randomized, double blind, placebo‐controlled	335^a^	6 to 12 years	Multicentre	Asthmatic children (6‐12 years), able to perform pulmonary function tests	Positive for respiratory syncytial virus, taking immunosuppressive medication (excluding steroids for asthma treatment), other uncontrolled medical condition, undergone antiviral treatment of influenza in previous 2 weeks	ITTI: n = 84 Per protocol: n = 73	ITTI: n = 95 Per protocol: n = 89	Primary: time to freedom from illness Secondary: area under the symptom score‐hour curve, proportion of patients with asthma exacerbations, changes in forced expiratory volume at 1 second	Serum influenza antibody titres and swabs for influenza viral culture	Within 48 hours of symptom onset	Oseltamivir 2 mg/kg twice daily as syrup OR placebo
Kaiser (2003)	Multicentre phase 3 trials in Northern and Southern Hemisphere influenza seasons from 1997 to 2000	Randomized, double‐blind, placebo‐controlled	769^b^	Older than 12	Multicentre	Healthy unimmunized adults and adolescents (13‐64 years), at‐risk patients (people 65 years or older and adults and adolescents with respiratory and/or cardiac conditions requiring regular outpatient care)		137^c^	157^c^	Primary: occurrence of lower respiratory tract complications requiring antibiotic intervention Secondary: hospitalizations, upper respiratory tract complications, overall antibiotic use	Virus isolation from nose and throat swabs or fourfold (or greater) rises in antibody titres	Within 36 hours of symptom onset	Oseltamivir 75 mg twice daily for 5 days OR placebo twice daily for 5 days
Lin (2006)	China, flu season of 2002 to 2003	Randomized, open‐label, controlled trial	56	Not stated	Nine university tertiary teaching hospitals in China	Patients with chronic respiratory diseases or chronic cardiac disease with confirmed influenza infection	Patients with high suspicion of bacterial infection, pregnant or nursing women, patients with history of alcohol or drug abuse, patients vaccinated for influenza within 12 months prior to start of study	ITT: 27	ITT: 29	Primary: duration and severity of illness Secondary: incidence of complications, antibiotic use, hospitalization and total medical cost	Virus isolation and serological analysis	Within 48 hours of illness onset	75 mg oral oseltamivir twice a day for five consecutive days OR routine symptomatic treatment in control group
Martin (2001)	‐	Randomized, placebo‐controlled, double‐blind, parallel‐group study	402	13 to 88 years	Two centres	Patients with chronic cardiac and/or respiratory disease with confirmed influenza infection		ITT: 199	ITT: 203	Duration of febrile illness, fever and other symptoms, viral shedding and complications	Positive virus culture or fourfold (or greater) rise in antibody titre	Within 36 hours of onset of influenza symptoms	Oseltamivir 75 mg twice daily for 5 days OR placebo twice daily for 5 days
Piedra (2009)	Same as in Table 1 above	1634^d^	3721^d^	Same as in Table 1 above						Frequencies of pneumonia, respiratory illnesses other than pneumonia, otitis media, and hospitalisation			
Singh (2003)	Multicentre	Pooled RCT	251^c^	13 to 97 years		Adolescents and adults (13‐97 years) with chronic respiratory and/or cardiac conditions presenting within 36 hours of influenza symptoms	Patients with clinically important chronic illness, known HIV infection, receiving immunosuppressants, history of alcohol or drug abuse, hospitalized	No subgroup data available	Primary: time to alleviation of a seven‐symptom cluster	Virus isolation of haemagglutination inhibition (HAI) antibody titration of baseline and convalescent serum samples	36 hours of symptom onset	Oseltamivir 75 mg twice daily for 5 days OR placebo twice daily for 5 days	

Abbreviations: ITT, intention‐to‐treat; ITTI, intention‐to‐treat infected.

^a^ 335 subjects were enrolled, one subject did not receive the study drug.

^b^ 769 represents “at‐risk” population which encompasses people with chronic cardio‐pulmonary disease and/or elderly individuals ≥65y as there is no subset data available specific to people with chronic cardio‐pulmonary disease.

^c^ Sub‐group data of patients with chronic respiratory and/or cardiac disease.

^d^ No subset data for children with cardio‐pulmonary diseases available to determine effectiveness outcomes so these values include children with ALL chronic medical conditions.

### 2.7 Risk of bias assessment

Risk of bias in selected randomized trials was assessed using Cochrane's Risk of Bias 2.0 tool.^17^ Risk of bias in nonrandomized studies was assessed with Cochrane's Risk of Bias in Nonrandomized Studies of Interventions (ROBINS‐I) tool.^18^ Overall risk of bias was deemed high if there was high risk of bias in ≥1 domain (confounding bias, selection of participants into the study, classification of interventions, deviations from intended interventions, incomplete outcome data, measurement of outcomes, and selective reporting within the studies), unclear if there was unclear risk of bias in ≥1 domain and low if there was low risk of bias across all six domains.

The quality of evidence across the different outcomes assessed in the systematic review was graded using the Grading of Recommendations, Assessment, Development, and Evaluations (GRADE) guidelines.^19^ The certainty of the evidence behind each outcome was assessed based on the study designs, risk of bias, inconsistency, indirectness, imprecision, publication bias, and participant size. Based on these factors, the overall quality of evidence was deemed very low, low, moderate, or high. The GRADEpro guideline development tool software was used to assist the synthesis of this data.^20^ Risk of publication bias was assessed qualitatively as part of the quality of evidence assessment. Quantitative assessment of publication bias was also carried out using funnel plots.

### 2.8 Synthesis of results and meta‐analysis

The main findings were summarized in a tabular format and a qualitative narrative synthesis of the results was undertaken. Meta‐analyses were performed to pool data from studies with sufficient information of the same outcome measures. Dichotomous data were analyzed using risk ratio (RR) and continuous outcomes were analyzed using weighted mean difference (MD). DerSimonian and Laird's random effects model was used to estimate the overall effect size with 95% confidence interval (CI). For continuous data, missing standard deviations were estimated from other summary statistics such as confidence intervals, standard errors, *t* values or *P* values. In studies where these values were missing, the corresponding authors were contacted and values reported as ranges. All statistical analyses were carried out using Review Manager (RevMan) software version 5.3.

## 3 RESULTS

### 3.1 Study selection

**FIGURE 1:**
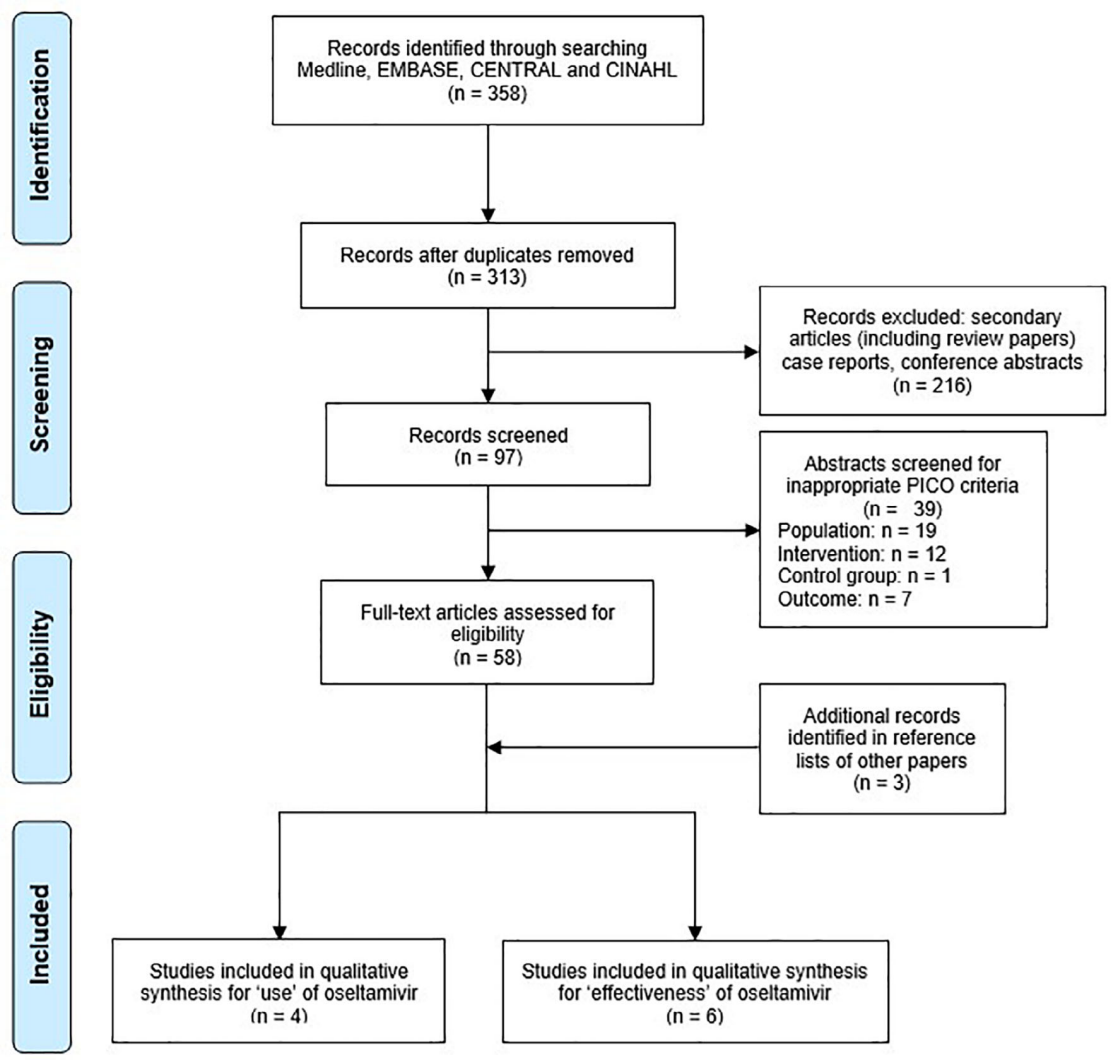
Study selection flowchart based on preferred reporting items for systematic reviews and meta‐analyses (PRISMA)^24^

The search generated 330 citations. After removing duplicates and studies that did not meet the eligibility criteria, 29 studies were assessed in full length. One study was excluded based on English language restriction.^21^ The reference lists of these studies were screened for additional studies and yielded three additional studies. The corresponding authors for two studies on effectiveness were contacted to obtain subset data specific to oseltamivir effectiveness in our high‐risk populations, however, these data could not be obtained.^22^, ^23^ Nine studies were included in the final analysis. One study had data on both effectiveness and rate of use of oseltamivir (Figure 1).

### 3.2 Participant characteristics

All the studies (n = 4) included to determine the rate of use of oseltamivir were conducted in patients aged <21 years. In the studies (n = 6) included to determine the effectiveness of oseltamivir, two were in children ranging from 1 to 17 years old,^11^ one did not state age range,^25^ and the remaining three studies were in participants older than 12 years old.

All the studies in our systematic review included broad populations, and so subset data on people with cardio‐pulmonary conditions were extracted according to our predetermined inclusion criteria. The subset data from three studies were from patients with asthma,^11^, ^26^, ^27^ and two were from those with cardio‐pulmonary conditions.^25^, ^28^, ^29^ Two studies defined their high‐risk population as those with chronic cardio‐pulmonary conditions and/or the elderly and as individual data were not available, the whole population was included.^30^, ^31^ In one study which included patients with any medical conditions, subset data could not be extracted and thus all participants were included as the majority of this population (85.0%) had cardio‐pulmonary conditions.^29^

### 3.3 Study characteristics

All four studies on the use of oseltamivir were observational studies, three being retrospective^27^, ^28^, ^29^ and one being prospective^23^ (Table 1). One study included ambulatory patients,^29^ two included hospitalized patients^26^, ^27^ and one included patients admitted to ICUs.^28^ Two of the studies only included patients prescribed oseltamivir within 24 hours of symptoms onset,^28^, ^29^ one study was within 48 hours of symptoms onset^27^ and the fourth study did not state treatment initiation in relation to symptom onset.^26^ All four studies were in patients with confirmed influenza infection. Influenza infection was confirmed through reverse transcriptase polymerase chain reaction (RT‐PCR)^26^, ^27^ or based on ICD‐9‐CM diagnostic codes of influenza.^28^, ^29^

Four of the six studies on effectiveness were randomized, double‐blind, placebo‐controlled multicentre trials^11^, ^22^, ^30^, ^31^ (Table 2). One study was a randomized open‐label trial.^25^ A retrospective study included to evaluate oseltamivir's effectiveness was also used to determine its rate of use.^29^ One study included patients prescribed oseltamivir within 24 hours of influenza diagnosis,^30^ two studies included patients prescribed oseltamivir within 48 hours of symptom onset^11^, ^25^ and three studies were patients within 36 hours of symptom onset.^22^, ^30^, ^31^ All studies on effectiveness were also in patients with confirmed influenza infection. Five studies confirmed influenza infection based on virus isolation from patient swabs and/or rises in serum influenza antibody titers^11^, ^22^, ^25^, ^30^, ^31^ while one study confirmed influenza based on ICD‐9‐CM diagnostic codes for influenza.^29^

### 3.4 Rates of use of oseltamivir

Rates of oseltamivir use varied between the four different studies from 25% to 100% (Table A1). Two studies were based on data from the USA and had prescribing rates of 25%^28^ and 31%.^29^ Subset data for people with chronic cardiac or respiratory disease were taken from these studies as their study populations were broader than required by our study. The study conducted in hospitals in Spain had 41% usage rate^27^ while Saudi Arabia had 100%.^26^ The subset data used from these two studies relevant to our inclusion criteria were patients with asthma.

### 3.5 Effectiveness of oseltamivir

**FIGURE 2:**
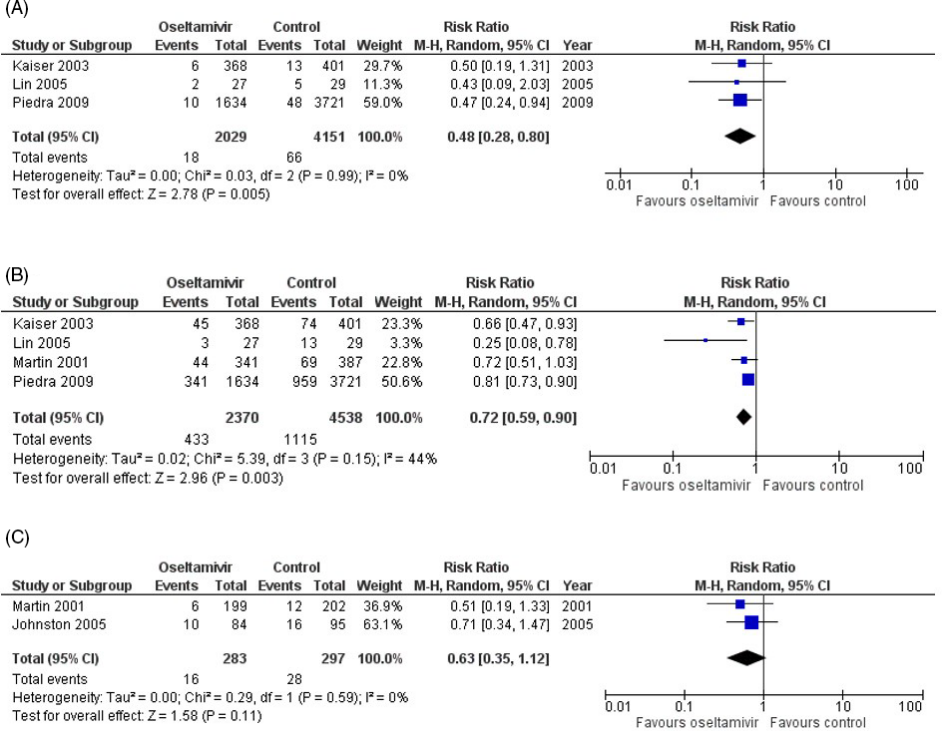
Forest plots comparing effectiveness of oseltamivir in reducing rates of, A, hospitalization, B, respiratory complications, C, asthma exacerbations in people with cardiopulmonary disease who were prescribed oseltamivir compared to those who were not

Meta‐analysis of the data suggests administration of oseltamivir for the treatment of confirmed influenza infection in patients with cardiopulmonary conditions compared to placebo reduced hospitalization rates significantly (RR = 0.48, 95% CI = 0.28‐0.80, I2 = 0%, Figure 2A). Rates of respiratory complications were also significantly less likely when comparing the two groups (RR = 0.72, 95% CI = 0.59‐0.90, 6908 patients, I2 = 44%, Figure 2B). There was no significant difference in rates of asthma exacerbations between the treatment and control group (RR = 0.63, 95% CI = 0.35‐1.12, 680 patients, I2 = 0%, Figure 2C). The absolute values, however, suggest a trend favoring oseltamivir when compared to control.

**FIGURE 3:**
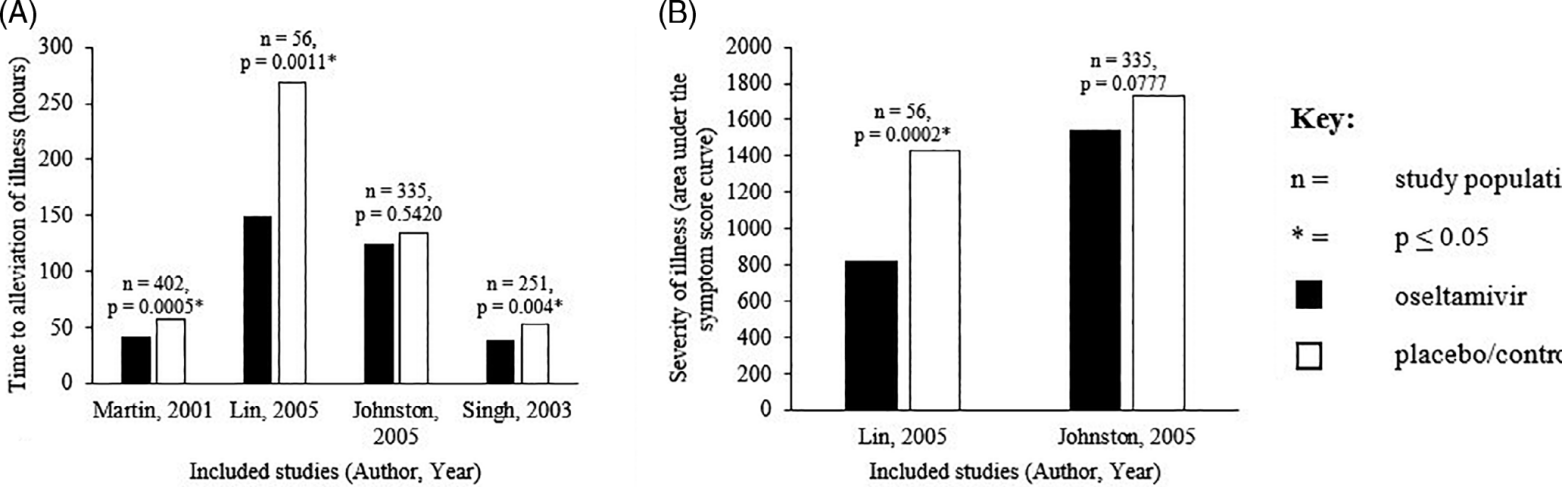
Effectiveness of oseltamivir in reducing, A, time to alleviation of illness (hours), b, severity of illness in people with cardiopulmonary disease who were prescribed oseltamivir compared to those who were not

Due to heterogeneity of data and inability to obtain subset data, a pooled analysis to determine time to alleviation of illness and severity of illness could not be performed. However, the absolute values indicated a reduction in the time taken to illness alleviation and reduced severity of illness.^11^, ^22^, ^25^, ^31^ Time to alleviation of illness ranged between 37.9 to 148.8 hours in the oseltamivir group and 40.8 to 268.8 hours in the placebo group (Figure 3A). The severity of illness was based on the area under curve symptom score in one study^25^ and the area under the symptom score‐hour curve in the second study.^11^ The severity of illness scores ranged from 817.1 to 1543.3 in the oseltamivir group and 1435 to 1731.3 in the placebo group, indicating greater severity of illness in placebo group (Figure 3B).

### 3.6 Risk of bias within studies

**FIGURE A1:**
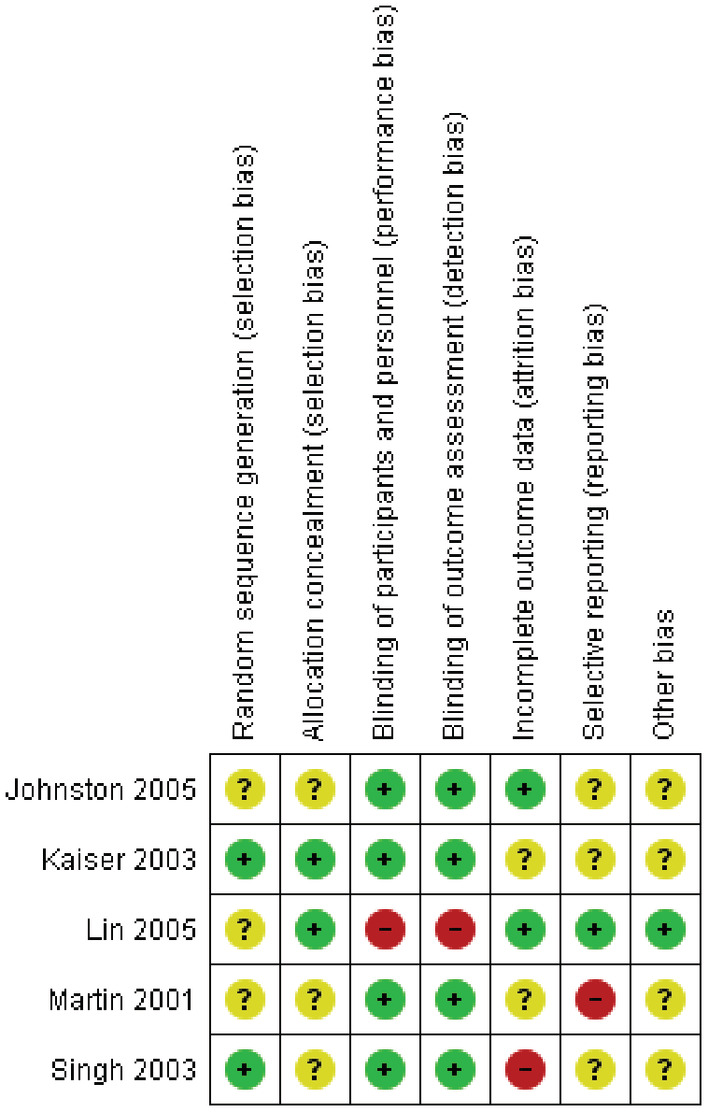
Risk of bias in randomised trials measuring effectiveness of oseltamivir (based on Cochrane RoB 2.0 tool)

**FIGURE A2:**
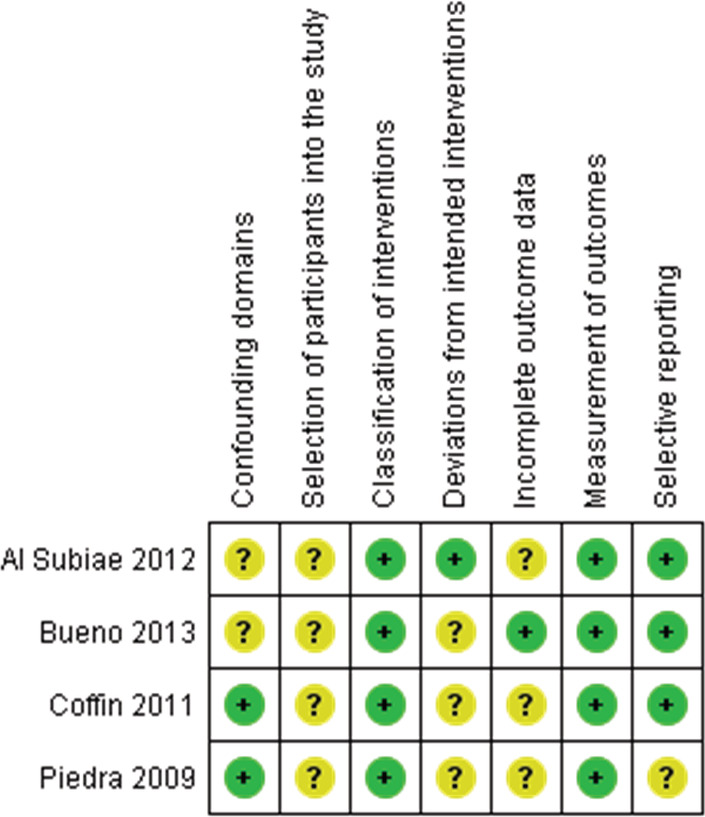
Risk of bias in nonrandomized studies measuring the rate of use of oseltamivir (based on ROBINS‐I tool)

**FIGURE A3:**
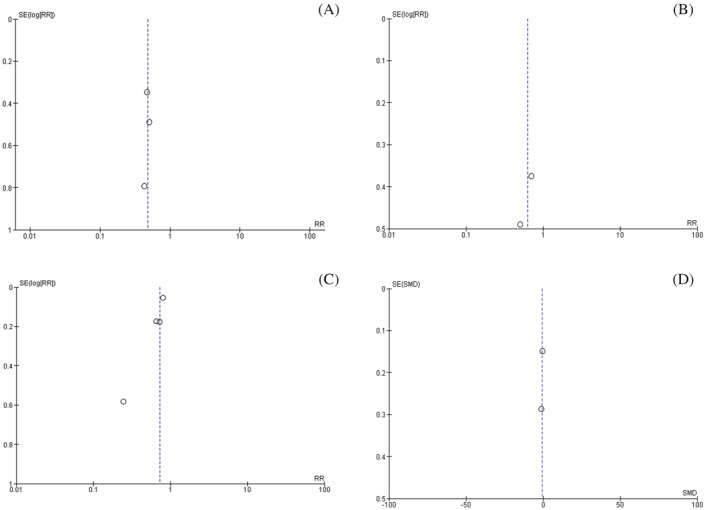
Funnel plots for: A, Rates of hospitalization, B, Rates of asthma exacerbation, C, Rates of respiratory complications, D, Severity of illness

There were five randomized controlled studies of which three had high overall risk of bias and two had unclear risk of bias (Figure A1). Four nonrandomized studies had overall unclear risk of bias (Figure A2). Risk of publication bias was deemed high based on qualitative assessment (Table A3) and quantitative evaluation of funnel plots (Figure A3).

### 3.7 Overall quality of evidence

The quality of evidence (Table A3) for the duration of illness and respiratory complications outcomes was judged to be high. The quality of evidence for hospitalisation and asthma exacerbation rate were deemed moderate while the certainty assessment for severity of illness outcome was low.

## 4 DISCUSSION

Our systematic review demonstrated that rates of oseltamivir use in people with cardio‐pulmonary conditions with influenza are suboptimal. The two studies from the USA from before the 2009 H1N1 influenza pandemic conducted in high‐risk children in both in‐patient^28^ and outpatient^29^ settings, had similar usage rates (25% and 31%). In contrast, the study which included hospitalized children with asthma in Saudi Arabia in the 2009 influenza season had an oseltamivir prescription rate of 100%.^26^ The only study with post‐2009 pandemic data included in the systematic review had prescription rates of 41% in children with asthma in Spain (2010‐2012).^27^ As the rate of use ranged between 20% and 40% outside of the pandemic years, it is possible that the high usage rate during the 2009 influenza^26^ season may have been due to the perceived risk associated with pandemic influenza leading to the implementation of pandemic strategies, making that year an outlier compared to usual prescribing practices.

Low rate of use could be associated with laboratory testing for influenza. Influenza is not routinely tested for and the lack of confirmatory laboratory data may impact the decision in prescribing oseltamivir despite its recommendation for empirical use in ILI.^3^, ^23^ The low usage rate may also be due to lack of data on its effectiveness, particularly regarding the target populations who would benefit most from treatment.^23^ Recent systematic reviews in both adult^32^ and paediatric^33^ non high‐risk populations have indicated modest benefit with oseltamivir. However, these studies were not in high‐risk populations for whom oseltamivir use is recommended by the WHO and for whom treatment may yield a greater benefit when compared to the general population. On the other hand, our systematic review which included only high‐risk patients suggests that oseltamivir was effective in reducing rates of hospitalization and respiratory complications in people with chronic cardiopulmonary disease who were prescribed oseltamivir when compared to those who were not. Our analysis suggests that despite the sub‐optimal use, oseltamivir is effective in improving health outcomes in this high‐risk population.

Our study demonstrated a 52% reduction in hospitalization rates and a 28% reduction in rates of respiratory tract infections in oseltamivir treated group when compared to placebo in patients with chronic cardiopulmonary disease. We also found that there was a trend suggesting that oseltamivir is effective in reducing the likelihood of asthma exacerbation. These three outcomes are generally indicators of a more severe clinical picture due to influenza infection, suggesting that oseltamivir use in these situations should be indicated to reduce morbidity in high‐risk patients.

Other studies have reported variable rates of effectiveness across these outcomes, such as a 34% reduction reported in otitis media incidence in children.^33^ This is in contrast to another systematic review which found no significant reduction in hospitalization rates, bronchitis, sinusitis, and otitis media in adults and children.^32^ However, these studies again did not focus on the high‐risk population for whom use is particularly recommended and could have higher beneficial impact.

Due to a lack of specific data in the studies which we required to conduct analysis, we could not perform meta‐analysis for two outcomes: duration of illness and severity of illness. Based on the available data, however, our systematic review found oseltamivir reduced duration of illness by 10.4 to 120 hours in the chronic cardio‐pulmonary population which suggests that there was a trend favoring a reduced illness duration. Other systematic reviews in the pediatric population without chronic conditions reported a similar reduction of 17.6 hours (CI 95% = 0.62‐34.7 hours)^33^ and 29 hours (CI 95% = 12‐47 hours).^32^ Similarly, we found that there was a trend in our data to show that the severity of illness was reduced in the oseltamivir group when compared with placebo.

### 4.1 Strengths and limitations

Our study is a comprehensive updated synthesis of available data on the use and effectiveness of oseltamivir in high‐risk population. This review evaluated the effectiveness of oseltamivir across multiple health outcomes relevant to decision making factors for clinicians.

Our study was limited to studies published in English, however, there was only one study excluded due to this language restriction.^21^ Despite extensive search strategy, there were only a few publications eligible for analysis, limiting the power of synthesized results. This highlights the need for more research in this specific population.

There was heterogeneity across the studies in terms of the study populations and study setting which limited pooled analysis of two of the health outcomes. In addition, the studies included in our systematic review had varied definitions of respiratory complications, we combined the upper and lower respiratory tract infections to standardize the outcome, which previous studies have also done.^34^ Despite each study having different parameters to define influenza‐related complications, oseltamivir demonstrated benefits over no treatment in reducing influenza associated complications including pneumonia and otitis media.^32^, ^33^

While some studies were focused on specific populations, others were very broad and subset data could not be obtained to quantitatively assess effectiveness for these studies. For instance, Johnston^11^ was focused on children with asthma, while Kaiser's^30^ “at‐risk” population included both elderly patients and/or people with cardio‐pulmonary disease, while Piedra^29^ included children with all chronic medical conditions in analyses for effectiveness. However, despite these variances, oseltamivir had improved outcomes when compared to control, suggesting a trend toward effectiveness.

In conclusion, our systematic review and meta‐analysis suggest oseltamivir is effective in reducing the severity of influenza illness in people with chronic cardio‐pulmonary disease, however, the rates of oseltamivir use in this high‐risk population are well below WHO recommendations. Further well‐designed studies evaluating the effectiveness and investigating the barriers to use of oseltamivir in high‐risk population are needed to better guide clinical management of influenza illness.

## CONFLICT OF INTEREST

The authors have no conflict of interest to declare.

## AUTHOR CONTRIBUTIONS

Conceptualization: Mei Chan, Louisa Owens, Adam Jaffe, Bernadette Prentice, Nusrat Homaira

Data curation: So‐Jung Shim

Formal analysis: So‐Jung Shim, Mei Chan

Investigation: So‐Jung Shim

Methodology: So‐Jung Shim, Mei Chan, Louisa Owens, Adam Jaffe, Bernadette Prentice, Nusrat Homaira

Project Administration: Mei Chan, Louisa Owens, Adam Jaffe, Bernadette Prentice

Supervision: Bernadette Prentice, Nusrat Homaira

Validation: Mei Chan, Louisa Owens, Adam Jaffe, Bernadette Prentice, Nusrat Homaira

Visualization: So‐Jung Shim

Writing—Original Draft Preparation: So‐Jung Shim, Nusrat Homaira

Writing—Reviewing & Editing: Mei Chan, Louisa Owens, Adam Jaffe, Bernadette Prentice, Nusrat Homaira

       All authors have read and approved the final version of the manuscript.

       Nusrat Homaira had full access to all of the data in this study and takes complete responsibility for the integrity of the data and the accuracy of the data analysis.

## TRANSPARENCY STATEMENT

Nusrat Homaira affirms that this manuscript is an honest, accurate, and transparent account of the study being reported; that no important aspects of the study have been omitted; and that any discrepancies from the study as planned (and, if relevant, registered) have been explained.

## Data Availability

Data available on request due to privacy/ethical restrictions. Please contact corresponding author to request data.

## Appendix

CINAHL:

01. Chronic lung disease

02. Chronic cardiac disease

03. Asthma

04. Cystic fibrosis

05. Oseltamivir

06. Tamiflu

07. Influenza

08. (S1 or S2 or S3 or S4) AND (S5 or S6) AND S7

CENTRAL:

01. (“chronic lung disease”)

02. (chronic cardiac disease)

03. (asthma)

04. (cystic fibrosis)

05. #1 or #2 or #3 or #4

06. (oseltamivir)

07. (Tamiflu)

08. #6 or #7

09. (influenza)

10. #5 and #8 and #9

MEDLINE:

01. Lung diseases/

02. Asthma/

03. Cystic fibrosis/

04. Bronchopulmonary dysplasia/

05. Heart diseases/

06. 1 or 2 or 3 or 4 or 5

07. Influenza, human/

08. Oseltamivir/ or oseltamivir.mp. or Tamiflu.mp.

09. 6 and 7 and 8

EMBASE:

01. Chronic lung disease/

02. Asthma/

03. Cystic fibrosis/

04. Lung dysplasia/

05. Heart disease/

06. 1 or 2 or 3 or 4 or 5

07. Oseltamivir.mp. or oseltamivir/ or Tamiflu.mp.

08. Influenza/

09. 6 and 7 and 8

**TABLE A1:** Rates of oseltamivir prescription reported in four different studies

Author (year)	Country, years	Oseltamivir (n = sample size)	No‐oseltamivir (n = sample size)	Total sample size (n = sample size)	Rate of use (%)
Al Subiae (2012)^a^	Saudi Arabia, July to December 2009	14	0	14	100
Bueno (2013)^a^ ^,^ ^b^	Madrid, September 2010 to June 2012	34	49	83	41
Coffin (2011)^c^	USA, October to April 2001 to 2007	56	172	228	25
Piedra (2009)^c^	MarketScan databases (Thomson Reuters, Cambridge, MA) from six influenza seasons (October 1‐March 31) 2000 to 2006	1401	3149	4550	31

^a^ No separate data for people with cardio‐pulmonary disease, however, there was sub‐group data for those with asthma which has been used here.

^b^ Criteria for treatment varied between hospitals (some hospitals had all hospitalised people treated, others only with risk factors).

^c^ This data represents the sub‐group data for people with cardiac conditions and respiratory conditions.

**TABLE A2:** Predeveloped data extraction template for use of oseltamivir and effectiveness of oseltamivir

Author (year)	Country, years	Study type, design	Study size	Age of participants	Clinical setting	Inclusion criteria	Exclusion criteria	Oseltamivir	No‐oseltamivir	Rate of use (%)	Confirmation of influenza infection	Treatment initiation	Treatment of oseltamivir group	Treatment of control group
		For example, double‐blind RCT, prospective cohort study		For example, children aged 0 to 17 years	For example, outpatient clinics			For example, 200	For example, 200		For example, PCR, viral culture	For example, within 48 hours of symptom onset	For example, 10 mg morning and night for 5 days	For example, placebo for 5 days

Author (year)	Country, years	Study type, design	Study size	Age of participants	Clinical setting	Inclusion criteria	Exclusion criteria	Oseltamivir	No‐oseltamivir	Outcomes	Confirmation of influenza infection	Treatment initiation	Treatment
		For example, double‐blind RCT, prospective cohort study		For example, children aged 0 to 17 years	For example, outpatient clinics			For example, 200	For example, 200		For example, PCR, viral culture	For example, within 48 hours of symptom onset	For example, placebo for 5 days

**TABLE A3:** Overall quality of evidence across the five outcomes for effectiveness of oseltamivir judged using the GRADEpro tool

Certainty assessment							No. of patients		
No. of studies	Study design	Risk of bias	Inconsistency	Indirectness	Imprecision	Other considerations	Oseltamivir	Control	Certainty
*Duration of illness*									
4	Randomized trials	Not serious^a^ ^,^ ^b^	Not serious	Not serious	Not serious	Publication bias strongly suspected strong association^c^	>299 days	>321 days	⊕⊕⊕⊕ HIGH
*Severity of illness*									
2	Randomized trials	Not serious^b^ ^,^ ^e^	Not serious	Not Serious	Serious^f^	Publication bias strongly suspected^c^	100	124	⊕⊕◯◯ LOW
*Hospitalisation rate*									
3	Randomized trials^g^	Not serious^h^	Not serious	Not serious	Not serious	Publication bias strongly suspected^c^	2029	4151	⊕⊕⊕◯ MODERATE
*Asthma exacerbation rate*									
2	Randomized trials	Not serious	Not serious	Not serious	Not serious	Publication bias strongly suspected^c^	283	297	⊕⊕⊕◯ MODERATE
*Respiratory complications*									
4	Randomized trials^g^	Not serious	Not serious	Not serious	Not serious	Publication bias strongly suspected strong association^c^	2370	4538	⊕⊕⊕⊕ HIGH

^a^ Duration of illness is a slightly more subjective outcome to measure (patients made diary entries of symptoms).

^b^ Lin, 2005: some concern for bias as study design was open‐label, not placebo‐controlled or blinded.

^c^ Martin, 2001, Kaiser, 2003 and Lin, 2005 were sponsored by Roche—a pharmaceutical company which produces oseltamivir. There were numerous trials on oseltamivir which have not been published, suggesting that its effectiveness may be overpublished.

^d^ Sum of the oseltamivir and no‐oseltamivir groups for Martin, 2001, Lin, 2005 and Johnston, 2006. No subset data available for Kaiser, 2003 for high‐risk population.

^e^ Severity of illness is a more subjective outcome to measure.

^f^ Not sufficiently large sample size (n < 400).

^g^ Majority of articles are randomized controlled trials, but includes one retrospective study (Piedra, 2009) which was assessed for bias using the ROBINS‐i tool.

^h^ All‐cause hospitalization rates were used (there may have been hospitalizations not related to influenza illness.
